# To the Profession

**Published:** 1882-07

**Authors:** J. Littlefield, Lee S. Smith

**Affiliations:** President


### TO THE DENTAL PROFESSION.

A meeting of dental dealers and manufacturers for consultation upon the interests of the trade in dental goods was held in Pittsburgh in February last. It was there decided to form a permanent organization, and this was consummated at a meeting held at Niagara Falls, June 21.

Through a misapprehension of the objects of this association by some members of the dental profession, the fear has been expressed that the intention was to combine for the purpose of raising prices, and in other ways to work injury to them.

It is the purpose of this paper to set forth the objects of the association, and to show that apprehensions of injury to the dentists by its operations are entirely unfounded.

Number 2 of the Articles of Association sets forth the purposes of the organization, as follows:

"The objects of this association are to reform abuses; to secure unity of action; to promote a friendly intercourse between its members; to avoid and adjust, as far as possible, differences and misunderstandings between them, and generally to advance the interests of the trade in dental goods in the United States."

This article is an honest and full expression of the objects of the Association. In their business relations the dentists, the dealers, and the manufacturers are a necessity, each to the others, and whatever really injures one class will eventually injure the others. Neither manufacturers nor dealers can legislate against the *true* interests of their customers without in the end injuring themselves.

The dealers in dental goods in this country have suffered for some years from wrong business methods. These have grown very largely out of a want of intimate acquaintance and intercourse, leading frequently to unnecessary and unwise competitions which largely increased expenses and losses by bad debts, and which, while benefiting a few customers, worked a positive injustice to many others.

Organization will tend to correct these evils, and, by periodical meetings, intercourse and discussion, information will be diffused, misapprehensions will be corrected, and the interests of all concerned will be promoted.

The dentists themselves long since decided that organizations with frequent meetings for the interchange of ideas and information are of inestimable value. It is believed that they will not be disposed to condemn, *a priori*, a plan for others which they have found so beneficial for themselves, but that they will be willing to accord to the dealers the privileges of organization without fear that the association will be used by its members against the interests of those who are in business their best friends.

The business of a dealer in dental goods must, of necessity, be of a limited character—very different from that of a dealer in dry-goods, groceries, iron or lumber; dealers in these articles have the entire population of the country for customers, while the number of *dentists* in the country is hardly more than twelve thousand.

One may be led to invest in luxuries for the table, in more fashionable garments, or more elegant furniture, without in any way reducing his *needs* for the future. Not so, however, with the business wants of the dentist; these are strictly limited to his practice, and if in any year he is led to buy more gold foil, rubber, teeth, instruments, etc., than his practice requires, his future purchases will be diminished in exact proportion. It is from forgetfulness of this fact, and from treating the wants of the dental profession as practically unlimited, that the wrong methods above referred to have mainly arisen, and they have wrought injury to both dealer and dentist.

I. The dealers have engaged very vigorously in a system of traveling far and wide, which has added largely to their expenses without proportionately increasing their profits; in some sections of the country they have crossed one another's tracks continually, often to the great annoyance of dentists, who have too frequently been called from patients to wait upon canvassers. Some have been led to give away a portion of their legitimate profits in order to make sales, and with this has been the inevitable tendency to press the sale of inferior goods in order to keep up profits. Some have been persuaded to give unwarrantable credits, and thus to incur unnecessary losses from bad debts.

The tendency has been in the direction of increasing expenses, increasing losses, constantly decreasing net profits, inferior quality of goods, and, in short, toward the degeneracy of the trade.

II. The dentists have been injured by this system in several ways:

1. It has wrought injustice to the many by the favors and concessions that have been given to the comparatively few. Cases were reported at Pittsburg of three dentists in the same town, having the same kind of practice, yet each paying a different price for the same goods; of a dentist who, by shrewdly setting three dealers to bidding against each other, purchased his office-chair, etc., at nearly twenty-five per cent. less than his neighboring competing dentists were paying. It was shown that the dentist who had the most time to canvass among the dealers, or who could succeed in causing two or more dealers to bid against each other, and the dentist who lived on the routes most frequently by travelers, were the ones who received favors in price and credit; while the confiding one who ordered of his dealer without bargaining, in the belief that he would be as well served as any, the busy dentist who had not time to shop, and those not so frequently visited by canvassers, were charged full rates, and therefore, as compared with the others, were treated unjustly.

These irregularities have, it is true, been limited, though of late the tendency has been to extend them, and it is undoubtedly true that far more than a majority of the dentists of the country have, by these practices, been placed at a disadvantage as compared with the minority.

The Dealers' Association proposes to correct this by adopting uniform rates for the same kind of goods, and treating all with equal fairness.

A schedule of discounts for large, strictly cash purchases has been adopted, and by giving all customers the benefit of it, the gross profits of the dealers will not be enhanced, and the dentists of the country, as a whole, will pay less for their supplies than under the old methods.

2. The competition on the road and the desire to do a larger business than is warranted by the nature of the trade have been to the dentist fruitful sources of debt, that in many instances has proved burdensome and embarrassing.

The temptation of long credit as an inducement to buy large bills in advance of any reasonable wants has been freely offered. Many dentists have thus been burdened through promises from salesmen of a credit "as long as convenient"—promises which the principals have not known of or consented to, and the result has been misunderstanding and ill-feeling.

Believing that, as a rule, it is no kindness to the average profesional man to induce him to incur debts beyond his needs for a moderate and reasonable time, the aim of the Association will be rather to offer inducements for cash transactions than to endeavor to make sales by offers of unreasonable credit. It is believed that the relations between dentist and dealer will be strengthened and that both will be benefited by the course.

3. The late methods of business have had the tendency to cause dentists on traveled routes to rely more upon travelers than upon their nearest local dealer. By encouraging him with his trade the dentist will enable his dealer to keep a better stock and to supply his wants as they arise, without the inconvenience of waiting for travelers.

One object of the Association is to enable the dealer to supply his own local trade at as low a rate as any others can, and, of course, more promptly.

4. A far more serious matter to the dentist than those above referred to is the inevitable tendency of an eager competition for cheapness toward depreciation in the quality of the goods offered. It has been truly said that "a competition for cheapness and not for excellence of workmanship is the most frequent and certain cause of the rapid decay and entire destruction of arts and manufactures."

No inducement that can be offered in the way of lower prices can in the slightest degree compensate for such depreciation. The greatest injury that can be inflicted upon the dentist, in view of the operations which he has continually to perform, is to supply him with inferior materials, appliances, and instruments. Most emphatically, in his case, "the best is the cheapest." There has been of late, as there must always be where a competition for cheapness exists, a tendency toward inferior goods.

The Association will, indirectly, and yet surely, operate to correct this, and to place competition on the nobler ground of contest for excellence in quality rather than for cheapness in price.

The association is not a combination for the purpose of establishing a schedule of prices for dental goods. Differences in quality and in prices have always existed and will continue to exist. Any attempt to harmonize these differences would obviously be impracticable. The various manufacturers of teeth, instruments, etc., will, as heretofore, make their own prices on their own products; but as manufacturers of dental supplies are also retailers, it is understood that whatever prices are established, or whatever alterations in prices are made, the facts shall be announced to the dealers, so that they shall have the privilege of selling as low as the manufacturer, and, on their part, it is understood that the manfacturer shall not be undersold on his own goods.

So far as prices are concerned, this is all there is in the organization. It does not make prices; it does not seek to control the manufacturers, nor to establish uniformity as to quality or price,—these points are left open to wholesome competition,—but it does seek to bring the dental trade to the one-priced system *on the same goods*,—a fair and just system which, once established, will give assurance to each customer that he is paying the same price for the same goods that his neighbor pays; and that without loss of time or temper in canvassing among different dealers.

Such a system cannot fail to commend itself to all fair men.

In brief, the *American Dental Trade Association* hopes, by correct business principles and methods, by associated action, by social intercourse and business conferences, to be an attraction and a benefit to its members; and it desires, by careful attention to the wants of the profession, and a constant effort to aid in the progress of dentistry, by fair dealing with every buyer, by an honest purpose to serve faithfully those who look to its members for supplies, by the assurance that the buyer who sends his order confidingly will surely receive as low rates as if he spent his time in bargaining, and that no competitor will receive special favors in prices, to commend itself to the confidence and esteem of the entire dental profession.

THE AMERICAN DENTAL TRADE ASSOCIATION.

*Address adopted.* J. LITTLEFIELD, *President.*
LEE S. SMITH, *Secretary.*

